# Preclinical Assessment of MEK Inhibitors for Malignant Peripheral Nerve Sheath Tumors Reveals Differences in Efficacy and Adaptive Response

**DOI:** 10.3389/fonc.2022.903177

**Published:** 2022-07-07

**Authors:** Yihui Gu, Wei Wang, Yuehua Li, Haibo Li, Zizhen Guo, Chengjiang Wei, Manmei Long, Manhon Chung, Rehanguli Aimaier, Qingfeng Li, Zhichao Wang

**Affiliations:** ^1^ Department of Plastic and Reconstructive Surgery, Shanghai Ninth People’s Hospital, Shanghai Jiao Tong University School of Medicine, Shanghai, China; ^2^ Department of Plastic Surgery, The Third Xiangya Hospital, Central South University, Changsha, China; ^3^ Department of Pathology, Shanghai Ninth People’s Hospital, Shanghai Jiao Tong University School of Medicine, Shanghai, China

**Keywords:** neurofibromatosis type 1, malignant peripheral nerve sheath tumors (MPNST), mek inhibitor, trametinib, target therapy

## Abstract

Malignant peripheral nerve sheath tumors (MPNSTs) are rare soft-tissue sarcomas refractory to standard therapies. Inactivation of *NF1* and subsequent upregulation of RAS/RAF/MEK/ERK signaling exist in the majority of MPNSTs. However, the lack of preclinical assessment of MEK inhibitors in MPNSTs hinders the clinical application as well as the development of combination therapy. To guide further clinical studies, we evaluated different MEK inhibitors in terms of efficacy, safety, and mechanism of adaptive response in treating MPNSTs. Using a MPNST tissue microarray, we found that p-ERK could serve as a biomarker for predicting the prognosis of MPNST patients as well as an effective therapeutic target. Through *in vitro* and *in vivo* experiments, we identified trametinib as the most potent MEK inhibitor for the treatment of MPNSTs. Mechanistically, reduced reactivation of the MAPK pathway and compensatory activation of the parallel pathways contributed to better efficacy. Our results provide a basis for the further clinical application of MEK inhibitors as single agents or combinational therapies.

## Introduction

Malignant peripheral nerve sheath tumors (MPNSTs) are aggressive soft tissue sarcomas arising in patients with neurofibromatosis type 1(NF1) or sporadically. MPNSTs are refractory to standard therapies (1, 2), and extended resection is currently the only potential curative management for MPNSTs, which is not suitable for advanced MPNST patients. In addition, the local recurrence rate remains high after surgical resection and traditional chemo/radio therapies show mild benefits (3–6). The overall 5-year survival rate has remained poor at only 16-52% for decades, mainly owing to the lack of effective treatments. Therefore, there is an unmet clinical demand to discover novel therapeutic strategies, particularly targeted therapies, to treat advanced MPNSTs.

Genomic profiling of MPNST cohorts revealed that mutation in Neurofibromin 1(*NF1)* occurred in 100% of NF1-associated MPNSTs and 82% of other types of MPNSTs. As a RAS GTPase–activating protein, inactivation of *NF1* leads to uncontrolled activation of RAS and subsequent upregulation of downstream signaling pathways including mitogen-activated protein kinase (MAPK), PI3K/Akt, and Ral-GEF pathways (7–12). Among these dysregulated signaling contributing to the occurrence and progression of MPNSTs, MAPK is the most well-studied signaling pathway which is critical for cell proliferation, differentiation, and survival, suggesting that this pathway is a promising therapeutic target in MPNST treatment. In addition, MAPK signaling pathway is also an effective therapeutic target for a variety of human cancers, including melanoma and NSCLC (13–15).

MEK inhibitors, such as trametinib and cobimetinib, are highly specific, non-ATP competitive, small-molecule inhibitors of MEK1/2. MEK inhibitors have shown promising therapeutic efficacy in RAS-driven tumors including plexiform neurofibroma (pNF), the precancerous lesion of MPNSTs (13, 16–20). In clinical trials, the MEK inhibitor selumetinib led to clinical responses in up to 70% of children with inoperable pNF and subsequently gained FDA approval for this indication (17, 21). However, the efficacy of MEK inhibitors in the treatment of MPNSTs remains controversial. Currently, the research related to MEK inhibitors mainly focused on the development of effective combinational strategies. Several combinational therapies including MEK inhibitors in combination with mTOR inhibitors, MET inhibitors, and others were evaluated in preclinical studies and clinical trials (22–24). However, clinically sufficient combinational therapies still do not exist.

We found that the efficacy of different MEK inhibitors as single agents and the mechanism underlying the difference in treatment response are poorly investigated in preclinical studies. In addition, the low incidence of MPNSTs makes the recruitment of clinical trials extremely difficult. These deficiencies and challenges hinder the implications of MEK inhibitors in MPNST therapy. Therefore, a comprehensive preclinical evaluation of MEK inhibitors is necessary to guide further clinical studies.

In this study, we evaluated different MEK inhibitors in terms of efficacy, safety, and mechanism of adaptive response in treating MPNSTs. We assessed the expression and clinical relevance of MAPK pathway in MPNST patients using a tissue microarray. The efficacy and safety of different MEK inhibitors were evaluated *in vivo* and *in vitro*. In addition, the mechanism of adaptive response in 8 MEK inhibitors was also explored. This study provided a basis for the further clinical application of MEK inhibitors as single agents or combinational therapies.

## Materials & Methods

### Cell Lines and Reagents

The NF1^+/-^ Schwann cell line (sNF02.3 2λ) was obtained from the American Type Culture Collection (ATCC). NF1-associated (ST88-14, T265, S462, and S462TY) and sporadic (STS-26T) MPNST lines were kindly granted by Prof. Vincent Keng and Prof. Jilong Yang (**Table S1**). All MPNST cell lines were maintained in high-glucose DMEM supplemented with 10% fetal bovine serum (FBS) and 1% penicillin/streptomycin in a 37°C, 5% CO_2_ incubator, sNF02.3 2λ cells were cultured under the same conditions with 1% L-glutamine added.

Reagents and antibodies used in this article are described in **Supplementary Table S2**.

### Immunohistochemical Staining

A tissue microarray (TMA) was constructed using 57 paraffin-embedded MPNST specimens obtained from 49 patients who underwent surgical treatment at Shanghai Ninth People’s Hospital, Shanghai Jiao Tong University School of Medicine in Shanghai, China. IHC staining was performed as previously described (25). The assessment of TMA IHC staining was accomplished by two qualified pathologists independently. The score was assigned according to the proportion of positive cells within carcinomatous areas so that no staining, <25% of malignant cells staining, 25%-50% of malignant cells staining, >50% of malignant cells staining were recorded as 0, 1, 2, 3. Clinical information was collected through electronic medical records and telephone follow-ups. Twenty-eight of 49 patients were lost to follow-up and were not able to be contacted through multiple methods.

### Cell Line-Based Assays

A Cell Counting Kit-8 (CCK-8) assay was implemented to assess cytotoxicity. A total of 1*10^3^ or 3*10^3^ cells per well were seeded into 96-well culture plates and treated with 0.1% DMSO or the indicated drug. After 72 h or 96 h, 10 µL CCK-8 solution (Dojindo, Japan) dispersed in 90 µL DMEM was added per well to measure the 450 nm OD value after a 2 h incubation. Percentage cell viability was calculated as 100% × (OD of drug-treated cells - OD of background control)/(OD of untreated cells - OD of background control). The IC50 of indicated drugs was calculated by Prism 8.4.0 using [inhibitor] vs. normalized response – Variable slope analysis.

Annexin V–FITC and propidium iodide (PI) assays were implemented to detect cell apoptosis. MPNST cells were seeded into 6-well culture plates and treated with DMSO or the indicated MEKi for 24 h or 48 h. Afterwards, MPNST cells were stained with annexin V–FITC and PI at room temperature in the dark for 15 minutes, followed by analysis using a flow cytometer (CytoFLEX LX, Beckman Coulter, Shanghai) equipped with CytExpert software (Beckman Coulter, Shanghai).

A cell cycle assay was implemented to monitor the cell cycle. MPNST cells treated as described above were fixed with cold 70% ethanol, stained with PI/RNase solution, and analyzed on a flow cytometer as described above.

### Western Blot Analysis

Cells were lysed in RIPA buffer (Beyotime, China) with protease and phosphatase inhibitor cocktails (Beyotime, China). Proteins separated by SDS-PAGE were transferred onto PVDF membranes and blocked in 3% bovine serum. Afterwards, membranes were incubated overnight in primary antibody solution at 4°C, followed by incubation with an HRP-conjugated secondary antibody for 1 h at room temperature. Band signals were detected using an Amersham Imager 600 (General Electric Company, Boston, MA, United States), and quantification was performed using ImageJ software after normalization to GAPDH.

### Xenografts

Four-week-old male NOD-SCID IL-2 receptor gamma-null mice were purchased from Shanghai Model Organisms Co., Ltd. (Shanghai, China). A total of 5×10^6^ S462TY cells suspended in 100 µL phosphate-buffered saline was engrafted subcutaneously into the armpit of each mouse. Tumor volume (based on caliper measurements) was calculated by the modified ellipsoidal formula: tumor volume = 1/2 length* width^2^. When tumors reached a volume of approximately 100 mm^3^, mice were randomly assigned to treatment with vehicle (n=4), trametinib (0.3 mg/kg, daily gavage, n=4), TAK-733 (2.4 mg/kg, daily gavage, n=4) or selumetinib (15.2 mg/kg, 2 times daily gavage, n=4). After 3 weeks of treatment, mice were sacrificed, and grafts were removed, weighed, and photographed. Grafts were stained with p-ERK, Ki-67, c-MYC, and cleaved Caspase 3 for histological analysis according to standard protocols.

### Statistics

Data are presented as the mean ± standard error of the mean (SEM) or standard deviation (SD). Statistical analysis was conducted using Prism 8.4 (GraphPad Software, San Diego, CA). Statistical analyses were performed using the student’s t-test, the log-rank test, and the Pearson correlation analysis. P-values < 0.05 were considered to indicate statistical significance, and asterisks (*) are used to indicate significant differences between two specified groups. * indicates a P-value < 0.05, ** indicates a P-value < 0.01, *** indicates a P-value < 0.001.

### Ethics Approval and Consent to Participate

The study was approved by the Ethics Committee of Shanghai Ninth People’s Hospital, Shanghai Jiao Tong University School of Medicine, and informed consent was achieved from patients under institutional reviewer board protocols.

## Results

### High Expression of p-ERK Correlates With Dismal Prognosis in MPNSTs

**Figure 1:**
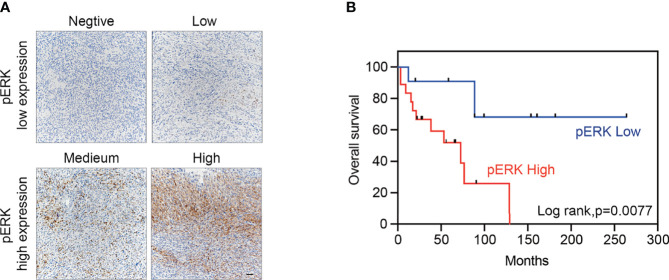
High expression of p-ERK correlates with dismal prognosis in MPNSTs. **(A)** Typical images of p-ERK in MPNST tissues following immunohistochemical staining, scored as 0 (negative), 1 (low positive), 2 (medium positive), and 3 (high positive). Scale bar = 50 μm **(B)** Kaplan–Meier curves for cumulative survival according to the p-ERK level in the MPNST cohort with available follow-up data (n = 29, p = 0.007 by the log-rank test). Negative and low positive were defined as low expression, while medium and high positive were defined as high expression.

To evaluate the expression of MAPK pathway in MPNSTs, we performed IHC staining of p-ERK using a TMA containing 57 MPNST specimens (**Table S3**). The levels of p-ERK in tumor tissues were classified as score 0 (negative, 20.8%), 1 (low positive, 28.3%), 2 (medium positive, 32.1%) or 3 (high positive, 18.9%) (**Figure 1A**). The expression of p-ERK was positively correlated with that of p-MEK, Ki-67, and CD34 (**Table 1**; **Figures S1A–C**). Analysis of clinicopathological correlations indicated that MPNST patients with high p-ERK expression had a shorter overall survival duration than the p-ERK low expression patients (p = 0.007, **Figure 1B**).

**Table 1:** Pathological parameter with p-ERK expression.

Markers	Correlation coefficients	95% confidence interval	p-value
p-MEK	0.5519	0.3358 to 0.7128	0.0001^***^
CD34	0.3695	0.1156 to 0.5781	0.0055^**^
Ki67	0.4105	0.1629 to 0.6094	0.0019^**^

**p<0.01, ***p<0.001 (Pearson correlation analysis, n= 55)

### Screening 8 MEK Inhibitors in MPNST Cells Revealed Differences in Efficiency and the Adaptive Response

**Figure 2:**
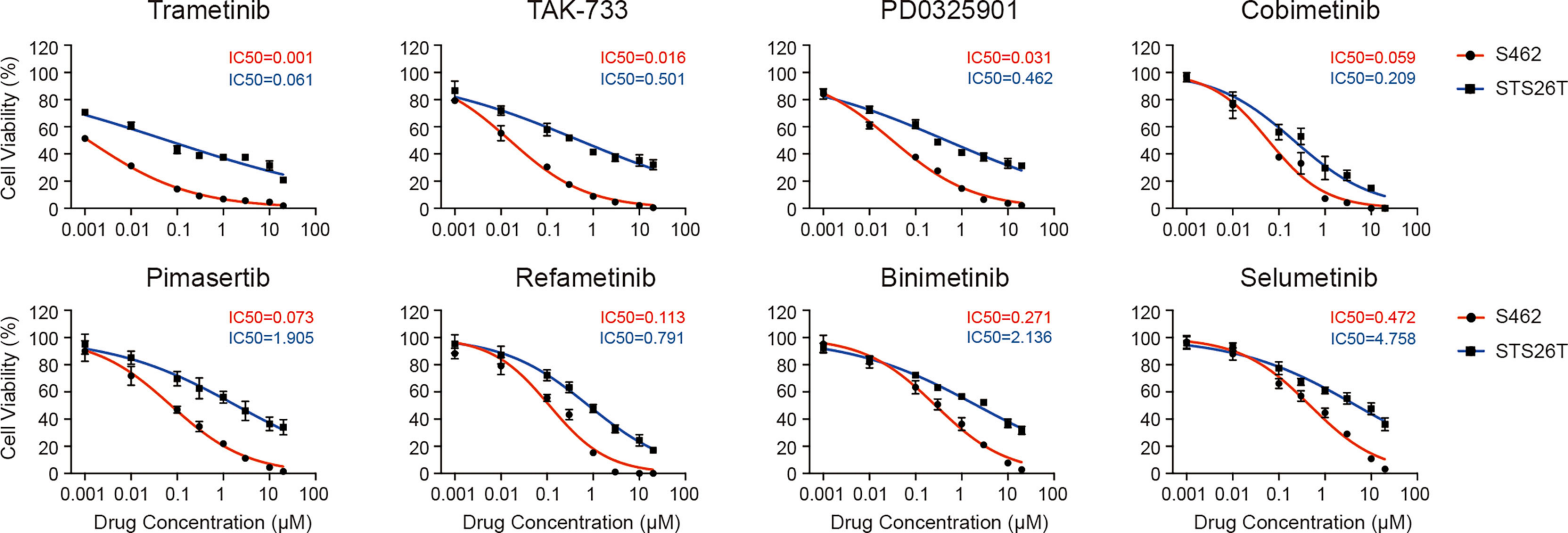
Different efficacy of MEK inhibitors in reducing the viability of MPNST cells. Cell viability of MPNST cell line S462 (the NF1-deficient human MPNST cell line) and STS26T (the NF1-wild-type human MPNST cell line with BRAF V600E and PTEN loss) after treated with MEK inhibitors for 96 h.

After identifying p-ERK as a potential therapeutic target for MPNSTs, we screened 8 different MEK inhibitors, trametinib, TAK-733, PD0325901, cobimetinib, pimasertib, refametinib, binimetinib and selumetinib, to investigate their efficacy in MPNSTs. We performed cell viability assays in S462 cells with loss of heterozygosity (LOH) at the NF1 locus. While STS26T with the oncogenic V600E mutation in BRAF served as a control since the BRAF V600E mutation has been shown to be associated with resistance to MEK inhibitors (26). The cell viability curves and corresponding IC50 values of these two cell lines are presented in **Figure 2**. Trametinib and TAK-733 were the most efficient due to having the lowest IC50 values. At the same time, the IC50 values of Trametinib and TAK-733 in S462 were significantly lower than those in STS26T.

**Figure 3:**
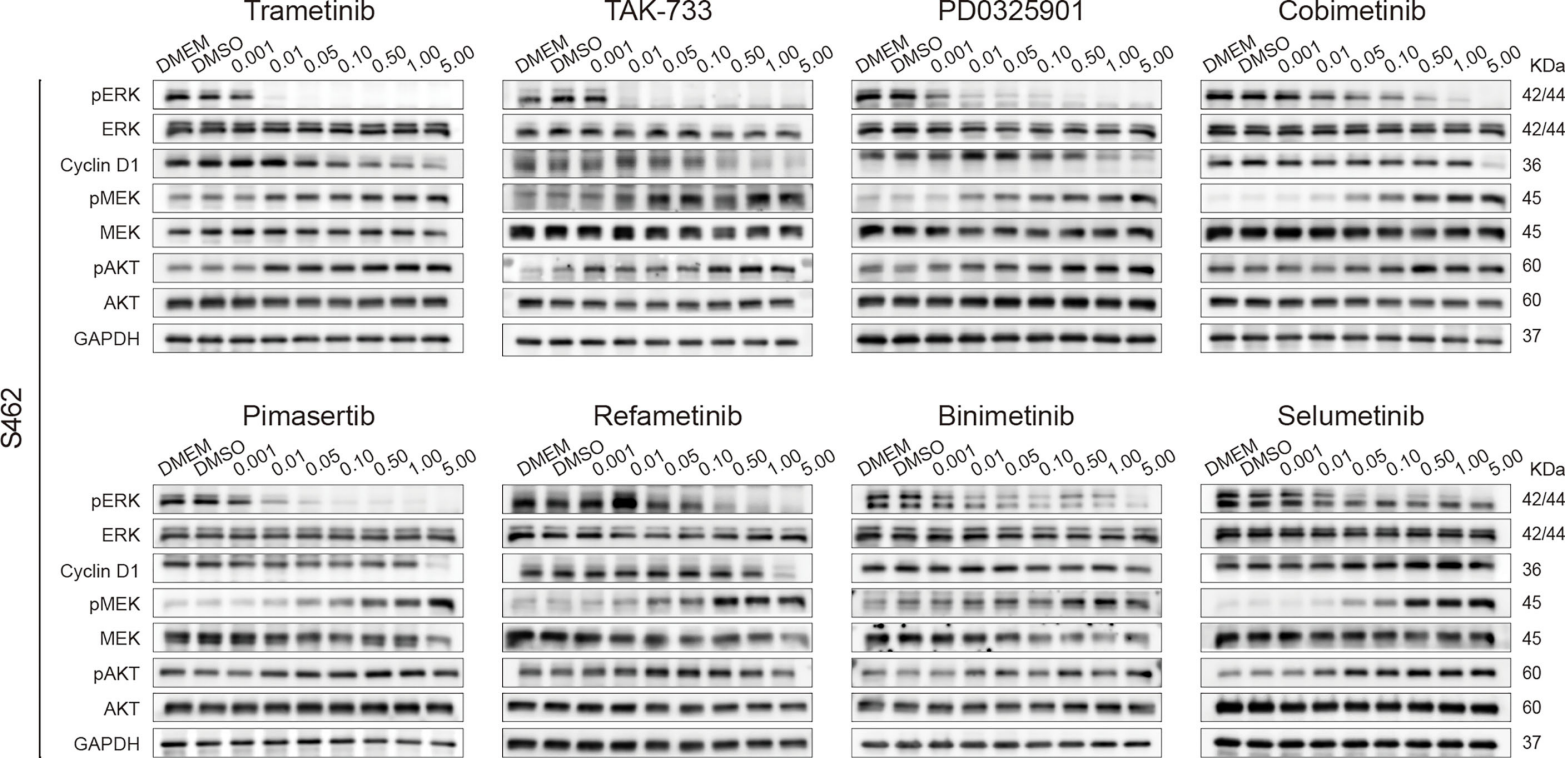
Screening 8 MEK inhibitors in MPNST cells revealed differences in adaptive response. Expression of p-ERK, ERK, cyclin D1, p-MEK, MEK, p-AKT, AKT, and GAPDH in S462 cells treated with different MEK inhibitors at the indicated concentrations for 24h.

To explore the mechanism underlying the difference in efficacy, we next assessed the alterations in protein expression, including p-ERK and p-MEK in the MAPK signaling pathway, the downstream MEK/ERK effector cyclin D1, and p-AKT in the parallel PI3K/AKT pathway. P-ERK levels decreased with increasing concentrations of MEK inhibitors. Trametinib and TAK-733 were the most potent inhibitors, as p-ERK was not detected in S462 cells following 24 h of treatment with 0.01 μM. Selumetinib was the least effective in reducing p-ERK levels. A similar variation tendency in cyclin D1 expression was observed. As demonstrated in previous studies, MEK inhibition could reactivate MAPK signaling or upregulate parallel signaling pathways, particularly, the PI3K pathway, resulting in reduced efficacy of MEK inhibitors (27). It was also observed in S462 cells that compensatory increases in p-MEK and p-AKT occurred after 24 h of MEK inhibitor treatment. Trametinib and TAK-733 induced upregulation of p-MEK less obvious than other MEK inhibitors (**Figure 3**, **Figure S2**).

### Inhibition of p-ERK Induced Cell Cycle Arrest and Apoptosis in MPNST Cells

**Figure 4:**
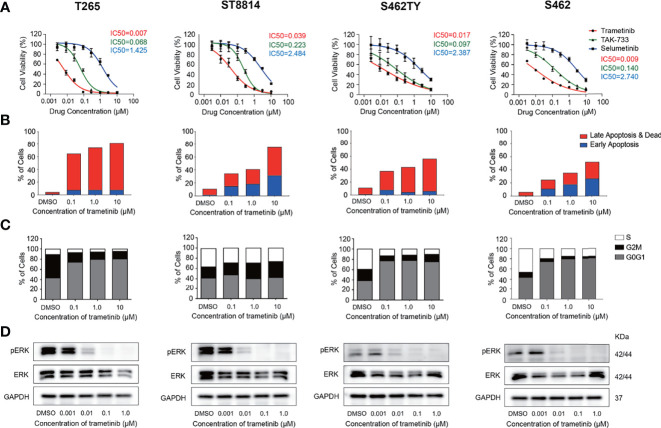
Inhibition of p-ERK induced cell cycle arrest and apoptosis in MPNST cells. **(A)** Cell viability of MPNST cell lines T265, ST8814, S462, S462TY, and Schwann cell line 02.3 2λ treated with MEK inhibitors for 72h. **(B)** apoptosis ratios of MPNST cell lines treated with MEK inhibitor trametinib for 48h. **(C)** Cell cycle and **(D)** p-ERK/ERK expression levels of MPNST cell lines treated with MEK inhibitor trametinib for 24h.

We next explored the efficiency and safety of MEK inhibitors in a variety of MPNST cell models. We selected the most potent MEK inhibitors trametinib and TAK-733, as well as selumetinib, the first FDA-approved MEK inhibitor for the treatment of pediatric PNFs, for further investigations. Cell viability assays were conducted in 4 NF1-MPNST cell lines S462, T265, ST88-14, S462TY, and schwann cell line 02.3 2λ. As shown in **Figure 4A**, trametinib inhibited all the MPNST cells most efficiently with submicromolar IC50 values. In addition, MEK inhibitors trametinib, TAK-733, and selumetinib were more efficient in MPNST cell lines compared with healthy Schwann cell line 02.3 2λ (**Figure S3**).

Furthermore, alterations in cell proliferation, apoptosis, and p-ERK expression following trametinib treatment were also explored in these MPNST cells. All the MPNST cell lines showed a high apoptosis ratio after trametinib treatment in a time-dependent manner (**Figure 4B**, **S4**). Significant blockage of the G0/G1 phase was observed in S462, T265, and S462TY cells while blockage of the G2/M phase was observed in ST88-14 cells (**Figure 4C**). As shown in **Figure 4D** and **Figure S5**, MEK inhibitor trametinib induced a significant reduction of p-ERK expression in S462, T265, ST88-14, and S462TY at a concentration of 0.01μM. The p-ERK expression in 02.3 2λ cells was significantly inhibited only at a higher concentration of 0.1μM (**Figure S6**).

### MEK Inhibitors Significantly Inhibited the Growth of MPNST Xenograft Without Inducing Apoptosis

**Figure 5:**
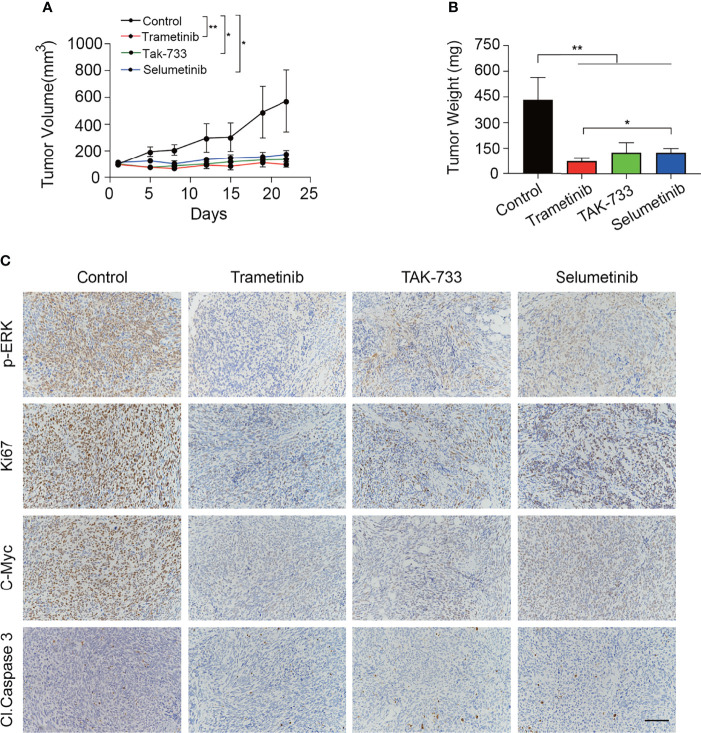
MEK inhibitors significantly inhibited the growth of MPNST xenograft without inducing apoptosis. **(A, B)** Tumor growth curves **(A)** and tumor weight **(B)** of mice treated with control, trametinib, TAK-733 or selumetinib for three weeks. Each point or column represents Mean ± SEM (n = 4). * Represents p < 0.05, and ** represents p < 0.01 compared with control group (Student’s t-test). **(C)** Representative images of p-ERK, Ki67, C-Myc, and Cl. Caspase3 immunohistochemical staining from the extracted tumor tissues in the four groups (scale bar = 100 μm).

The MEK inhibitors trametinib and TAK-733 and selumetinib were further evaluated *in vivo*. Following the successful establishment of S462TY xenografts, mice were gavaged with trametinib, TAK-733, or selumetinib for 3 weeks. As shown in **Figures 5A, B**, all the three MEK inhibitors reduced the tumor weight and tumor volume significantly. The inhibition in tumor volume persisted from the beginning to the end of administration with no obvious toxicity (**Figure S7**). Immunohistochemical analysis of the MPNST xenografts demonstrated that the three MEK inhibitors induced a robust decrease in p-ERK, Ki-67, and C-MYC at the protein level. However, no obvious alteration of cleaved Caspase 3 expression was observed (**Figure 5C**).

## Discussion

In this study, we investigated the efficacy of 8 different MEK inhibitors in treating MPNST using cell lines and xenograft models. We identified p-ERK as a biomarker for predicting the prognosis of MPNST patients as well as an effective therapeutic target. Moreover, we identified trametinib as the most potent MEK inhibitor for MPNST and demonstrated that the reduced reactivation of the MAPK pathway and activation of the parallel pathways contributed to better efficacy.

According to previous studies, genetic alterations including loss of NF1, EED, and SUZ12, have been demonstrated to activate the RAS/MAPK pathway in various human cancers including MPNSTs (7, 28, 29). Our findings also confirmed the overactivation of the MAPK pathway in MPNSTs as evidenced by the detection of p-ERK expression. In addition, our result first identified p-ERK as an indicator of a worse prognosis, indicating that the MAPK pathway could serve as a potential target in MPNSTs.

Inhibiting the RAS/MAPK pathway using MEK inhibitors has emerged as an effective strategy in a variety of cancers (30–34), as indicated in several clinical trials. However, the low incidence of MPNSTs added difficulties to the recruitment of clinical trials evaluating MEK inhibitors for MPNSTs. Therefore, we evaluated a panel of MEK inhibitors in various MPNST models and investigated the mechanism underlying the difference in efficacy. We included all the FDA-approved MEK inhibitors, and several potent MEK inhibitors proved to be efficient in clinical trials. Among the 8 inhibitors evaluated *in vitro*, trametinib was the most effective, likely from the significant reduction in p-ERK and downstream cyclin D1 levels. In addition, trametinib induced the lowest increase of p-MEK in S462 cells because it uniquely blocked Raf-dependent MEK phosphorylation, resulting in its potent efficacy in MAPK inhibition. Our *in vivo* experiments also confirmed the efficiency of MEK inhibitors. According to the results above, trametinib turned out to be the optimal choice for clinical application considering its excellent anti-tumor capacity *in vitro* and *in vivo*, as well as its mild reactivation of the MAPK pathway. As demonstrated by a recent case report, trametinib resulted in sustained complete response in an NF1 patient with recurrent and metastatic MPNST (35). In addition, trametinib has been approved by FDA for the treatment of malignant melanoma. Studies are being carried out to evaluate the effectiveness and to screen combinational drugs of MEK inhibitors in cancers including ovarian cancer and lung cancer (19, 32, 36–38).

Despite the potent efficacy of MAPK inhibition, trametinib caused significant activation of PI3K/Akt, a parallel pathway of MAPK signaling. It has been demonstrated in a variety of cancers that parallel pathways, especially the PI3K/Akt signaling pathway, could promote tumor growth and result in adaptive resistance after inhibition of the MAPK signaling (39, 40). Concurrent inhibition of MAPK and PI3K/Akt signaling using combinational therapies increased the efficacy of MEK inhibitors in multiple RAS-driven cancer models including MPNSTs (41, 42). Currently, a clinical trial is being carried out to test the effectiveness of selumetinib (AZD6244) and the mTOR inhibitor sirolimus in patients with unresectable or metastatic MPNST(NCT03433183).

Selumetinib is an FDA-approved MEK inhibitor that has shown excellent therapeutic efficacy in the treatment of pNF, the precursor lesion of MPNSTs. Compared to trametinib, selumetinib induced a more robust compensatory increase in p-MEK levels, indicating the occurrence of reactivation of the MAPK pathway, which might explain the less effective tumor-killing capacity. Wang et al. reported that in liver cancer, combinational therapy of sorafenib and selumetinib could increase the anti-tumor effectiveness through the synergistic inhibition of p-ERK (43). This combinational therapy might also improve the efficacy of selumetinib in MPNSTs.

It is also worth noticing that the MEK inhibitors inhibited the growth of MPNST xenografts without inducing tumor shrinkage or tumor cell apoptosis *in vivo*. It was inconsistent with the findings in Annexin V/PI assays showing that MEK inhibitors induced cell apoptosis significantly *in vitro*. It could be speculated that the tumor microenvironment including immune cells and extracellular matrix played an important role in limiting the efficacy of MEK inhibitors. Previous studies have suggested that microenvironment components are associated with drug resistance and are considered key obstacles that limit the efficacy of targeted therapy (44–49). For example, fibroblast-derived ECM abrogated anti-proliferative responses to BRAF/MEK inhibition in BRAFV600-mutated advanced melanoma and pharmacological targeting of key molecules could overcome drug resistance (50). Therefore, the role of the tumor microenvironment in limiting the efficacy of MEK inhibitors in treating MPNSTs and the underlying mechanisms deserve further investigation.

In conclusion, the variety of MEK inhibitors and the lack of evaluation of their efficacy in MPNSTs limited the research progress and clinical application of MEK inhibitors in MPNSTs. This study identified the most promising MEK inhibitor trametinib, for the treatment of MPNSTs and clarified the mechanism underlying the difference in efficacy. Our results provide a basis for the further investigation of MEK inhibitors for treating MPNSTs.

## Data Availability Statement

The original contributions presented in the study are included in the article/**Supplementary Material**. Further inquiries can be directed to the corresponding authors.

## Ethics Statement

The studies involving human participants were reviewed and approved by Ethics Committee of Shanghai Ninth People’s Hospital, Shanghai Jiao Tong University School of Medicine. The patients/participants provided their written informed consent to participate in this study. The animal study was reviewed and approved by Ethics Committee of Shanghai Ninth People’s Hospital, Shanghai Jiao Tong University School of Medicine.

## Author Contributions

YG, WW, YL, and HL generated and analyzed the data. ZG, CW, ML, MC, and RA assessed the TMA IHC staining and collected the clinical information. YG, WW, YL, and HL interpreted the results and wrote the manuscript. QL and ZW supervised the study and revised the manuscript. All authors contributed to the article and approved the submitted version.

## Funding

This work was supported by grants from National Natural Science Foundation of China (82102344; 82172228); Shanghai Rising Star Program supported by Science and Technology Commission of Shanghai Municipality (20QA1405600); Science and Technology Commission of Shanghai Municipality (19JC1413); Natural Science Foundation of Shanghai (22ZR1422300); “Chenguang Program” supported by Shanghai Education Development Foundation (SHEDF) (19CG18); Shanghai Municipal Key Clinical Specialty (shslczdzk00901); Innovative research team of high-level local universities in Shanghai (SSMU-ZDCX20180700); the Project of Biobank (YBKA201901) from Shanghai Ninth People’s Hospital, Shanghai Jiao Tong University School of Medicine.

## Conflict of Interest

The authors declare that the research was conducted in the absence of any commercial or financial relationships that could be construed as a potential conflict of interest.

## Publisher’s Note

All claims expressed in this article are solely those of the authors and do not necessarily represent those of their affiliated organizations, or those of the publisher, the editors and the reviewers. Any product that may be evaluated in this article, or claim that may be made by its manufacturer, is not guaranteed or endorsed by the publisher.
