# Supplementary material for: Preclinical Assessment of MEK Inhibitors for Malignant Peripheral Nerve Sheath Tumors Reveals Differences in Efficacy and Adaptive Response
Source: Front Oncol. 2022 Jul 7;12:903177. doi: 10.3389/fonc.2022.903177 (PMC9303010; doi:10.3389/fonc.2022.903177)
Supplement: Supplementary file 1 [file DataSheet_1.docx]

Supplementary Material

Table S1. Genomic alterations of the MPNST cell lines in current study

| Cell lines | History of NF1 | Mutations | Reference  (PMID) |
| --- | --- | --- | --- |
| S462 | YES | NF1 c.6792C>A; LOH  TP53 c.389G>C | 15207265  25810463 |
| S462TY | YES | NF1 c.6792C>A; LOH  TP53 c.389G>C | 18281493 |
| ST-8814 | YES | NF1 c.910 C>T exon skip; LOH | 1483704 1988828 |
| T265 | YES | n.a. | 9766530 |
| STS26T | NO | BRAF p.V600E/het  PTEN p.F144I/Y; p.F341I/Y; p.F514I/Y; LOH  TP53 ex4 del10/homozygous | 28556483; 16510576; 29854299 |

Table S2. Reagents and antibodies

| Product Name | Company | Cat.No. |
| --- | --- | --- |
| trametinib | MedChemExpress | HY-10999 |
| cobimetinib | MedChemExpress | HY-13064 |
| TAK-733 | MedChemExpress | HY-13449 |
| selumetinib | MedChemExpress | HY-50706 |
| pimasertib | MedChemExpress | HY-12042 |
| PD0325901 | MedChemExpress | HY-131295 |
| binimetinib | MedChemExpress | HY-15202 |
| Refametinib | MedChemExpress | HY-14691 |
| Cyclin D1 antibody | Abcam | ab134175 |
| Cl.Caspase 3 antibody | Abcam | ab32042 |
| CD34 antibody | Abcam | ab81289 |
| C-MYC antibody | Abcam | ab32072 |
| ERK1/2 antibody | CST | 4695 |
| p-ERK antibody | CST | 4370 |
| MEK1/2 antibody | CST | 4694 |
| p-MEK antibody | CST | 2338 |
| AKT antibody | CST | 4691S |
| p-AKT antibody | CST | 4060 |
| Ki67 antibody | CST | 9449 |
| GAPDH antibody | EASYBIO | BE0034 |

Table S3. Clinical parameter with pERK expression

|  | pERK expression | | *p*-Value |
| --- | --- | --- | --- |
|  | high | low |  |
| Gender |  |  |  |
| Male | 10 | 16 | 0.075 |
| Female | 17 | 10 |  |
| Age |  |  |  |
| <45 | 16 | 12 | 0.339 |
| >45 | 11 | 14 |  |
| Tumor size |  |  |  |
| T1 (<5) | 10 | 6 | 0.964 |
| T2 (5-10) | 4 | 11 |  |
| T3 (10-15) | 4 | 6 |  |
| T4 (>15) | 5 | 2 |  |
| Tumor site |  |  |  |
| Head and neck | 8 | 7 | 0.743 |
| Trunk | 8 | 9 |  |
| Limbs | 9 | 10 |  |
| NF1 |  |  |  |
| With | 11 | 9 | 0.474 |
| Without | 12 | 15 |  |

Pearson correlation analysis, n= 53


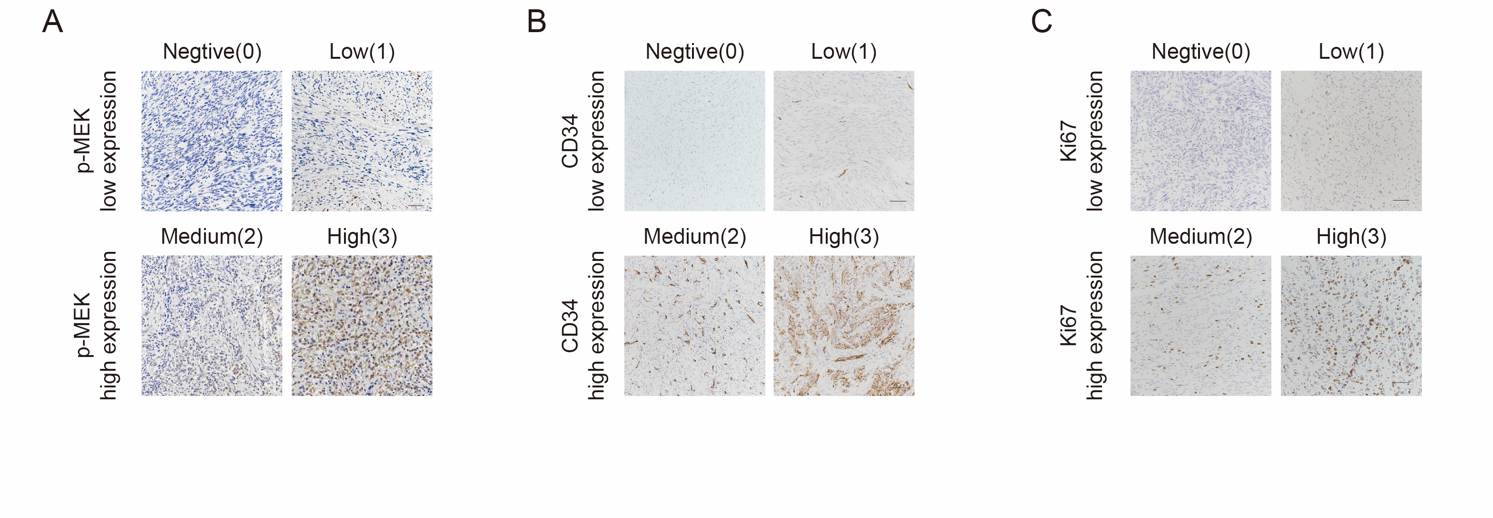


Figure S1. Typical images of pMEK (A), CD34 (B), Ki67 (C) in MPNST tissues following immunohistochemical staining, scored as 0 (negative), 1 (low positive), 2 (medium positive), and 3 (high positive). Scale bar = 50 μm


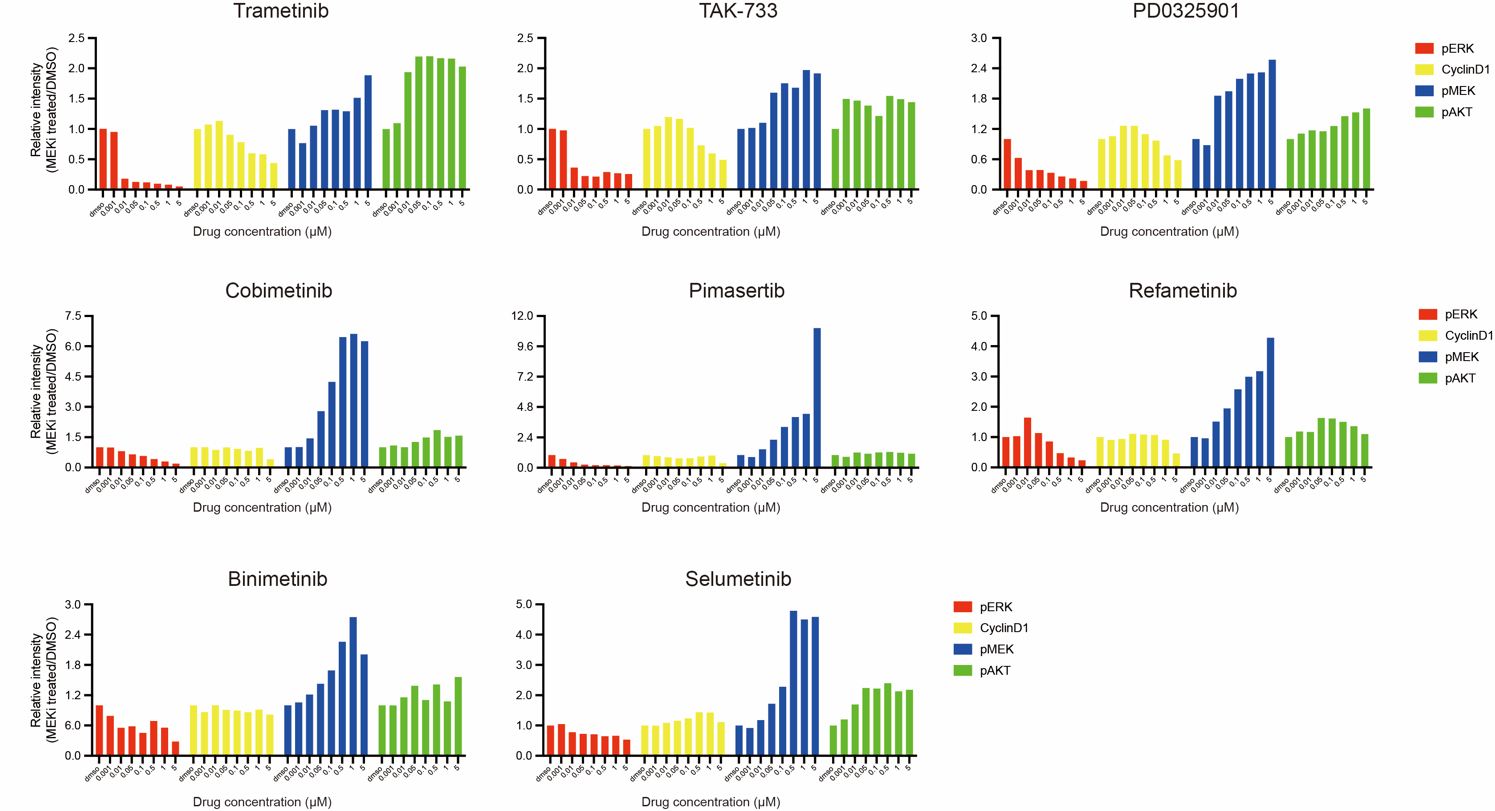


Figure S2. Relative intensity of pERK/ERK, CyclinD1/GAPDH, pMEK/MEK and pAKT/AKT in MPNST cell lines S462 treated with different MEK inhibitors for 24 h compared to DMSO-treated group.


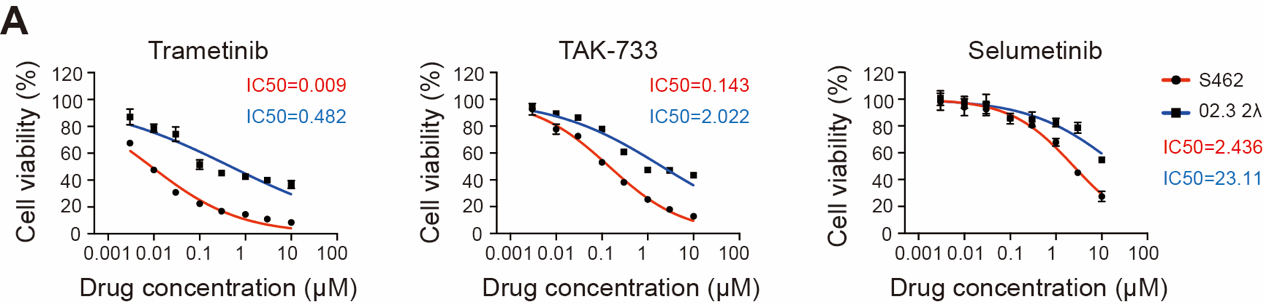


Figure S3. Dose-response curves and IC50 values of trametinib, TAK-733, and selumetinib at the indicated concentrations in S462 and 02.3 2λ cells. Data are represented as mean ± SD.


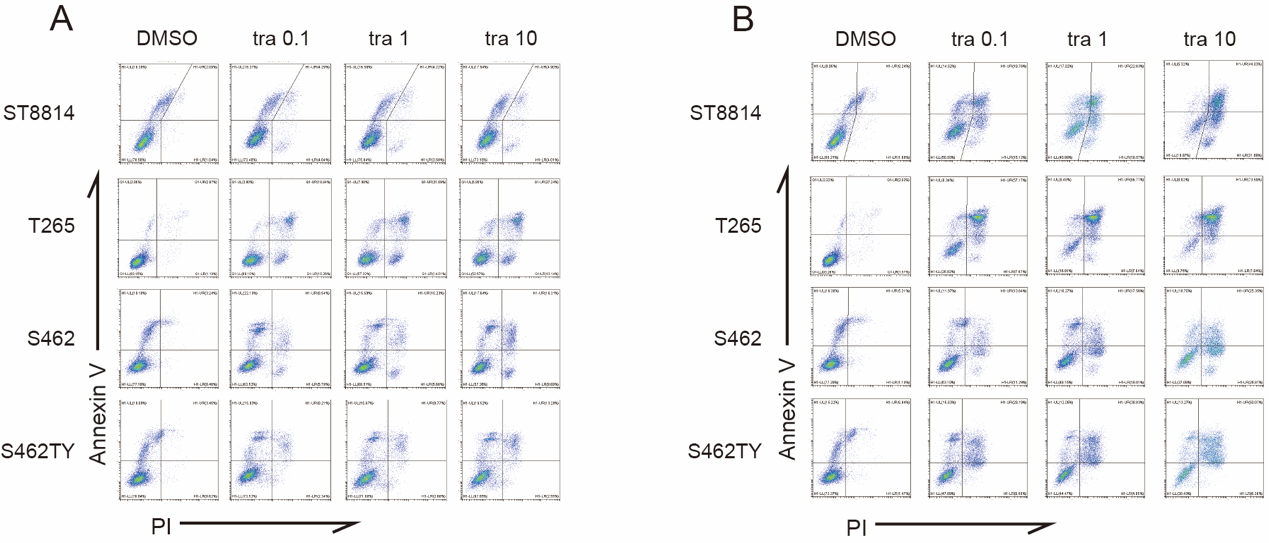


Figure S4. Apoptosis ratios of MPNST cell lines after treated with trametinib for 24 h (A) and 48 h (B).


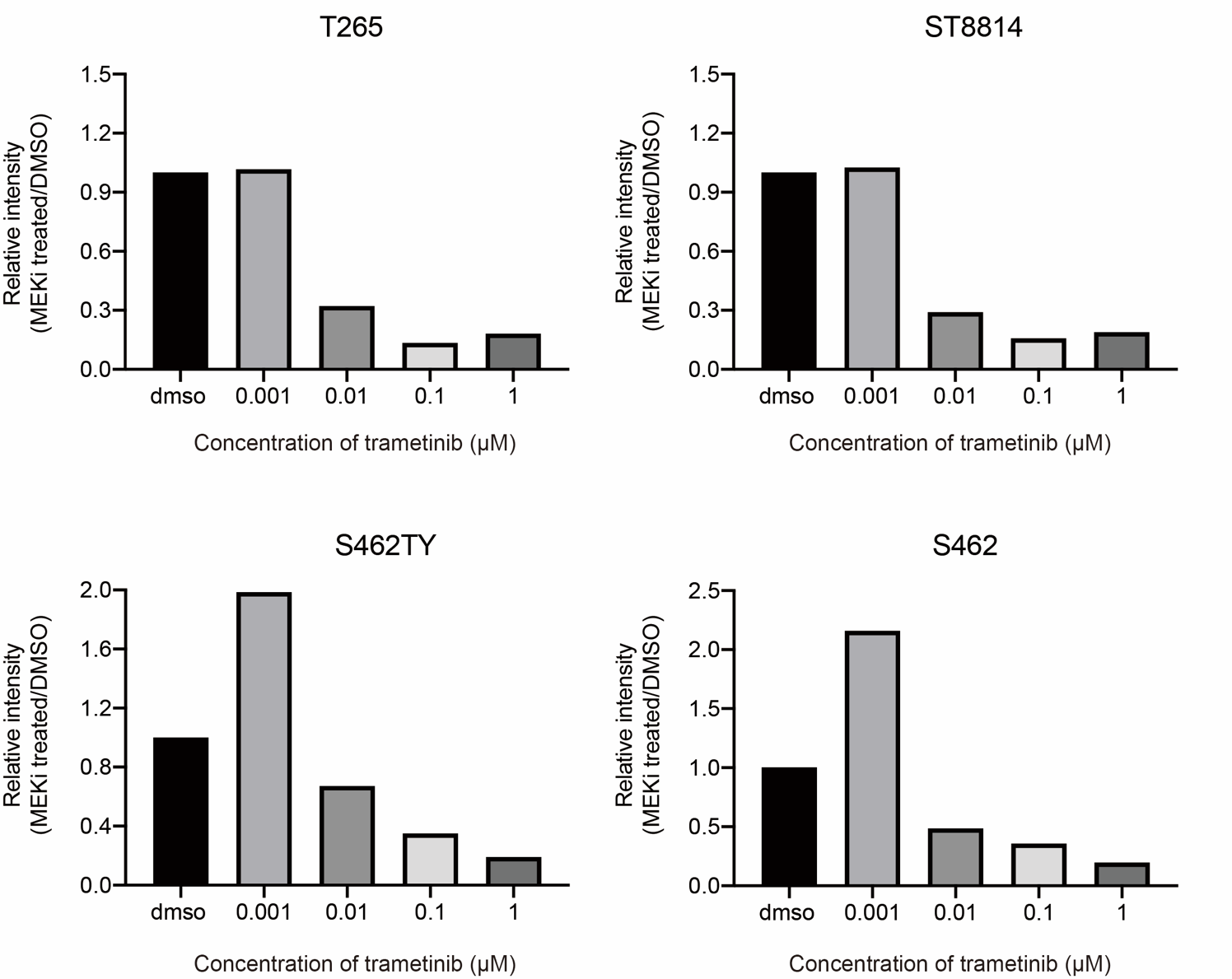


Figure S5. Relative intensity of p-ERK/ERK in MPNST cell lines (T265, ST8814, S462TY and S462) treated with MEK inhibitor trametinib for 24 h compared to DMSO-treated group.


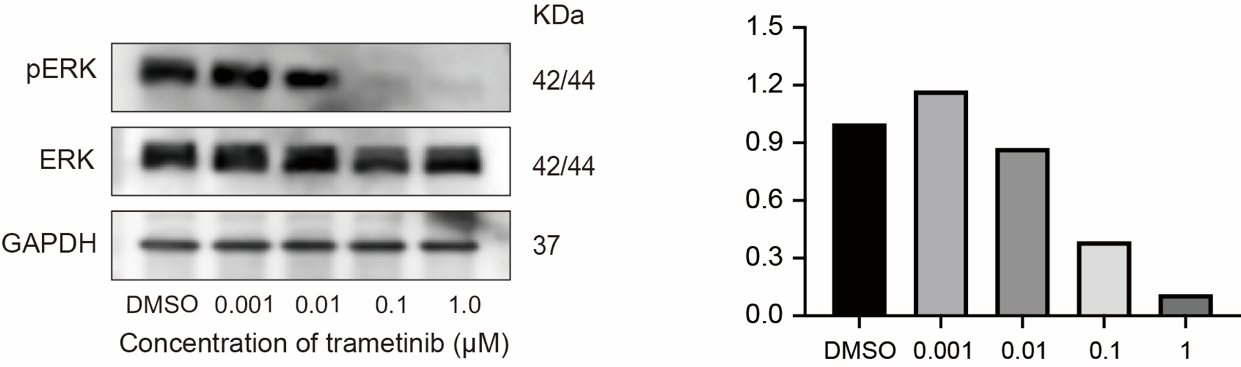


Figure S6. p-ERK/ERK expression levels of 02.3 2λ cells treated with MEK inhibitor trametinib for 24h.


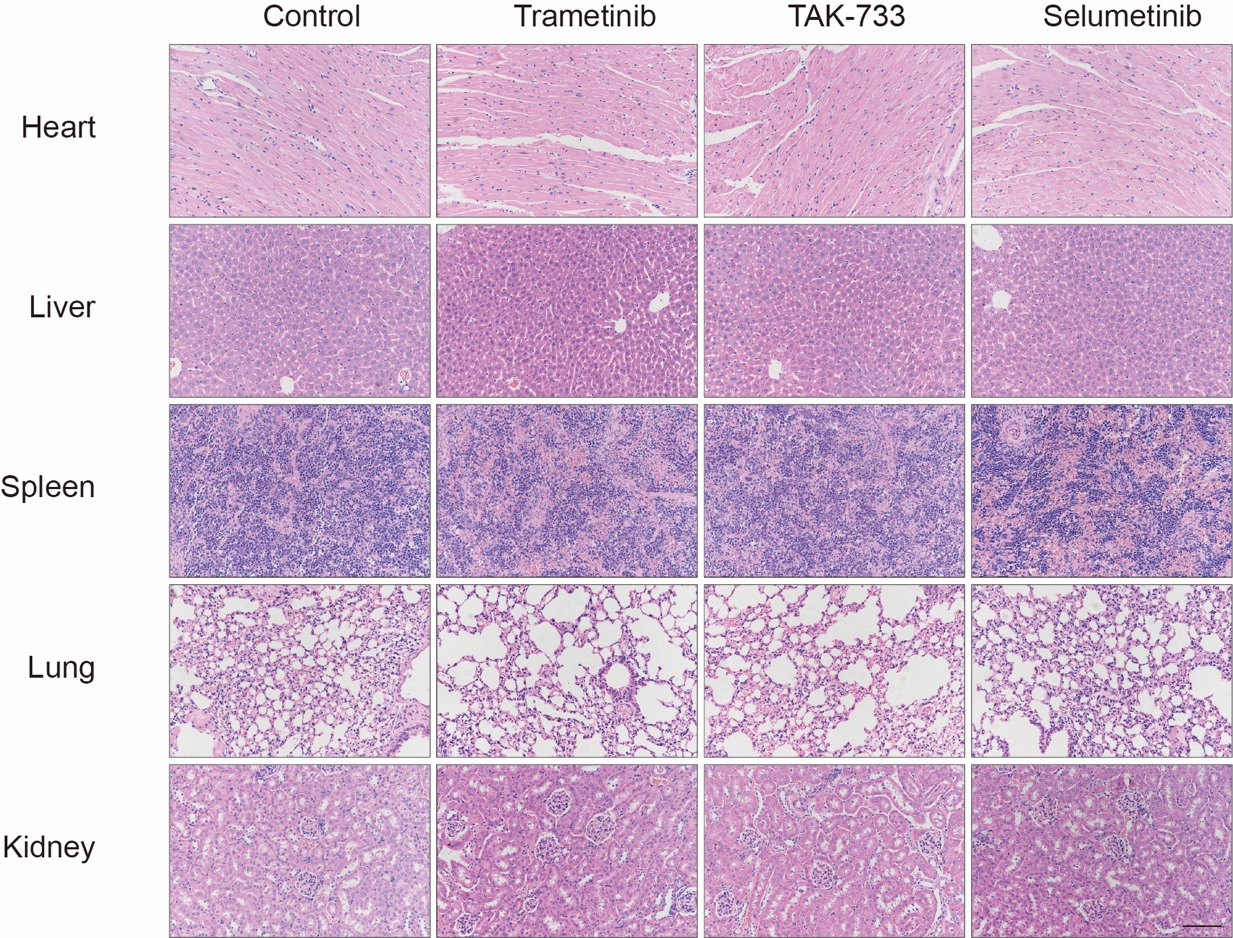


Figure S7. H&E staining of major organs (heart, liver, spleen, lungs, and kidneys) in MPNST-bearing NSG mice following treatment for 3 weeks with control, trametinib, TAK-733 or selumetinib. Scale bar =100 μm
